# Association between diet quality and food waste in Canadian families: a cross-sectional study

**DOI:** 10.1186/s12937-020-00571-7

**Published:** 2020-06-09

**Authors:** Nicholas Carroll, Angela Wallace, Kira Jewell, Gerarda Darlington, David W. L. Ma, Alison M. Duncan, Kate Parizeau, Michael von Massow, Jess Haines

**Affiliations:** 1 Department of Family Relations and Applied Nutrition, University of Guelph, 50 Stone Road East, Guelph, ON N1G 2W1 Canada; 2 Department of Mathematics and Statistics, University of Guelph, 50 Stone Road East, Guelph, ON N1G 2W1 Canada; 3 Department of Human Health and Nutritional Sciences, University of Guelph, 50 Stone Road East, Guelph, ON N1G 2W1 Canada; 4 Department of Geography, Environment and Geomatics, University of Guelph, 50 Stone Road East, Guelph, ON N1G 2W1 Canada; 5 Department of Food, Agricultural and Resource Economics, University of Guelph, 50 Stone Road East, Guelph, ON N1G 2W1 Canada

**Keywords:** Diet quality, Food waste, Family studies, Adults, Children

## Abstract

**Background:**

Higher diet quality has been associated with greater amounts of food waste among adults in the United States. This study aims to build on previous work by examining the association between diet quality and food waste, as assessed using detailed waste audits, among a sample of Canadian families.

**Methods:**

This cross-sectional study used data from 85 Canadian families with young children. Parent and children diet quality was assessed using the Healthy Eating Index-2015 (HEI-2015), calculated from 3-day food records. Household food waste was measured using detailed waste audits conducted over multiple weeks and these data were used to calculate daily per capita food waste. Linear regression was used to explore the association between parent and child HEI-2015 scores and daily per capita total avoidable and unavoidable food waste, as well as daily per capita avoidable and unavoidable food waste in the following categories: 1) fruits and vegetables, 2) milk, cheese and eggs, 3) meat and fish, 4) breads and cereals, 5) fats and sugars.

**Results:**

Parent HEI-2015 scores ranged from 37 to 92 (out of 100) and 81% of parents’ diets scored in the “Needs Improvement (51-80)” category. Parent and child diet quality scores were significantly correlated (r = 0.61; *P <* 0.0001) and 82% of children’s diets scored in the “Needs Improvement” category. On average, households produced 107 g of avoidable food waste and 52 g of unavoidable food waste per person per day. Fruits and vegetables were the highest contributor for both avoidable and unavoidable food waste. Both parent and child HEI-2015 scores were not significantly associated with total daily per capita avoidable or unavoidable food waste. However, parent HEI-2015 scores were positively associated with daily per capita avoidable fruit and vegetable waste (Unstandardized β = 1.05; 95%CI: 0.11, 1.99; *P* = 0.03) and daily per capita unavoidable fruit and vegetable waste (Unstandardized β = 0.60; 95%CI: 0.03, 1.17; *P* = 0.04), after adjusting for household income.

**Conclusion:**

This is the first study to explore the association between diet quality and food waste using detailed waste audits. Future research should explore effective strategies towards improving diet quality while simultaneously reducing food waste, especially of fruits and vegetables.

## Background

In Canada, it is estimated that approximately 40% of all food produced is lost or wasted [1]. Waste from households account for nearly half of the avoidable food waste, which is valued to be $10.4 billion worth of food being discarded each year [2]. Thus, it is important to explore characteristics and behaviours associated with food waste at the household-consumer level.

A previous study led by Conrad and colleagues [3] found that higher quality diets were associated with greater amounts of food waste among American adults. Given the majority of food waste in households come from fruits and vegetables [4], it is possible that individuals who have a higher diet quality cook and prepare more of these foods, which could lead to more unavoidable (inedible portions, like stems or peels) waste through the preparation process. It is also possible that health conscious individuals purchase more healthful foods, such as fruits and vegetables, but fail to eat them before they spoil as a result of poor storage or planning, which could lead to more avoidable (edible portions) waste. Understanding how diet quality is associated with household food waste can provide insight on which families to target to reduce food waste as well as potential strategies to minimize food waste.

Food waste was measured by Conrad et al. using estimates derived from the United States Department of Agriculture (USDA) Loss-Adjusted Food Availability data series. Use of these estimates may lead to an overestimation of food waste [5]. In addition, these estimates from aggregate data fail to provide accurate assessments of avoidable and unavoidable food waste [6]. To better understand how diet quality is associated with food waste, research that uses waste audits to capture detailed and accurate measures of food waste at the consumer level is needed.

Research indicates that food waste habits may differ across contexts, suggesting the importance of conducting region- or context-specific studies. A study by Secondi and colleagues [7] describes a multi-level analysis on household food waste behaviour across 27 European countries. Although some generalizations emerged from the study, there were notable cross-country differences – for instance, individuals residing in either the Czech Republic, Malta and Estonia were shown to have significantly lower amounts of food waste as compared to individuals residing in Denmark, Sweden, the Netherlands and Ireland [7]. Limited research has explored household food waste within the Canadian context using detailed waste audits [4, 6, 8] and no studies have examined the association between diet quality and food waste in Canada.

The present research aims to address this gap by examining the association between diet quality and food waste, assessed using detailed waste audits, among a sample of Canadian families. Based on the results by Conrad et al. [3], we hypothesize that diet quality will be positively associated with both avoidable and unavoidable household food waste.

## Methods

### Study design

This cross-sectional study used data from the Family Food Skills Study, which is a family-based study designed to understand associations between parental food literacy and dietary intake among families with young children. Families were eligible if they had at least one child between 2 and 5 years of age (in 2017) and 2–8 years of age (in 2018) and resided in Guelph-Wellington area of Ontario. In addition, parents had to be comfortable speaking and reading in English as well as having no prior food or nutrition training (e.g. Registered Dietitian, Chef, or Culinary Student). Families were recruited through social media, events in the community and local childcare centres. Data collection took place over a 4-week period. Initial home visits were conducted prior to data collection to provide families with an overview of the study and to obtain written, informed consent. After the data collection was completed, final home visits were completed to collect study material and to provide the grocery gift card incentive.

**Fig. 1:**
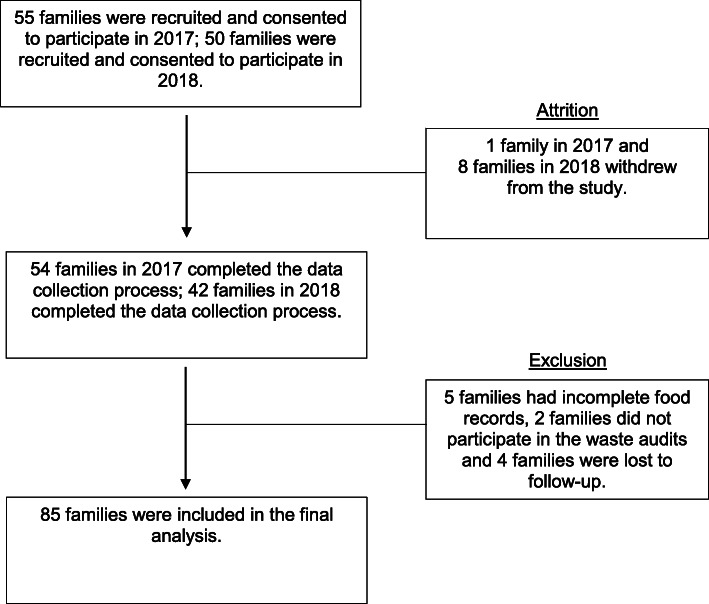
Flowchart of the Family Food Skills Study (FFSS) families in 2017 and 2018

Recruitment and data collection occurred in two waves. The first wave occurred in August–September 2017 and the second occurred in August–September 2018. Of 55 families enrolled in the study in the 2017 wave, 1 family withdrew, and 7 families were excluded due to missing data. A total of 50 families enrolled for the study in the 2018 wave, but 8 families dropped out and 4 families were excluded due to incomplete sets of data. Thus, our final analytic sample included 85 families. The primary reason for withdrawal from the study was scheduling conflicts which made it difficult to complete the data collection. In families with more than one child in the target age range, the oldest child was chosen to participate in 2017; whereas, in 2018, the child with the nearest birthdate was selected to participate in the study. While some families had two parents participate in the study, only data from parent 1 (the first parent to sign up) were included in these analyses. All details are illustrated in Fig.1.

### Variables

#### Diet quality (Healthy Eating Index-2015)

Parents completed 3-day food records, which included details about all food and beverages consumed on two weekdays and one weekend day for themselves and for their participating child. Our study protocol did not specify that food recording had to be consecutive days. Detailed instructions on how to complete the food records were provided during the initial home-visit. Parents were provided supplementary documents to help complete the food records including a guide for estimating portion sizes and an example of a complete food record. Completed food records were entered into the Food Processor Nutrition Analysis Software version 11.6.441 (ESHA Research, Salem, OR, USA) by a trained research assistant. A second research assistant then checked the entered data to ensure accuracy.

Parent and child diet quality was assessed using the Healthy Eating Index-2015 (HEI-2015). Under the supervision of a Registered Dietitian (RD), the food records were manually examined by research assistants for HEI moderation and adequacy components, which are expressed relative to energy intake, i.e., as densities, and converted to HEI equivalents. Sodium and fatty acid intakes were obtained from ESHA Food Processor Nutrition Analysis Software and were also used as adequacy and moderation components. Added sugars were manually calculated through retrieving information from designated food labels. The research assistants then calculated HEI-2015 scores based on average intake over the three days. The HEI-2015 is a validated measure of diet quality for individuals aged 2 years and older [9]. Scores range from 0 to 100, with higher scores indicating better diet quality. The HEI has been adapted for use with Canadian populations [10]. Since the version of ESHA used data based on the USDA recommendations – the HEI-2015 was most appropriate for this analysis.

#### Daily per capita food waste

Waste audits were conducted over a four-week period in 2017 and a three-week period in 2018 to determine daily per capita food waste. The shorter audit period in 2018 was driven by logistical constraints (i.e., availability of auditors and a holiday long-weekend, which can change waste behaviors). Research assistants collected all three waste streams (garbage, recycling, and organic bins) on the day the family would normally have their waste collected by the municipality and delivered the material to a waste sorting facility each week for four (2017) and three (2018) consecutive weeks. Individual food items were categorized and weighed (in grams) separately and sorted into the following six categories: fruits and vegetables, milk, cheese and eggs, meat and fish, breads and cereals, fats and sugars, and other (e.g. primarily coffee grounds). The other category was not used in the statistical analysis for this study. These categories were taken from the Waste and Resources Action Programme (WRAP) Household Food Waste Collection Guide [11].

Subsequently, each item was categorized as avoidable, unavoidable or possibly avoidable food waste. Avoidable was defined as food being discarded that is edible (e.g. bread, apples) [12]. Food items in this category typically could have been eaten if managed better. Unavoidable was defined as food that is not edible (e.g. banana peels, coffee grounds) [12]. Possibly avoidable were items that can be eaten or prepared in different ways depending on the individual (e.g. potato skin, apple skin). Some food items were categorized as unidentifiable (e.g. waste that could not be recognized as belonging to one of the six food categories) or unknown (e.g. waste that could not be identified as a particular food but could be identified as belonging to a food group category). Unidentifiable/unknown foods made up approximately 11% of the total food waste. The weights of these unknown/unidentifiable foods were assigned proportionally to the six food categories to develop estimates of total waste output. For example, if a household had “carrot” food scraps that constituted 15% of the household’s known vegetable weight, the “carrot” category would receive 15% of that household’s “unknown vegetable” category. Lastly, some items were identified as non-food organics (e.g. teabags, paper towel). There was no unavoidable waste in the breads and cereals, as well as, fats and sugars food category; therefore, these were not included in the analysis on daily per capita unavoidable food waste. The total mean weights of the 2017 and 2018 subsamples were compared using a Mann-Whitney test and the sub-groups were found to be appropriate for combined subsequent analysis.

For the purpose of this analysis, avoidable and unavoidable food waste were reported as a daily average by dividing the weekly amount of waste by 7 days. Subsequently, a per capita amount of food waste was generated by dividing the daily average by number of family members in the household.

#### Household income

Household income was measured and used as a covariate in the analysis as it has been observed to be associated with household food waste [13], as well as diet quality [14]. A single item was used to assess household income: “What is the total annual income of your household before taxes? Your household income includes income from you and anyone who lives with you who depends on the same income. Be sure to include income from all sources, such as salary and wages, child support, interest, public assistance and pensions.” Response options included: “Less than $10,000, $10,000 to $19,999, $20,000 to $29,999, $30,000 to $39,999, $40,000 to $49,999, $50,000 to $59,999, $60,000 to $69,999, $70,000 to $79,999, $80,000 to $89,999, $90,000 to $99,999, $100,000 to $149,999, $150,000 or more, I don’t know, and I am not comfortable answering this question”. The mid-point for each quantitative category was calculated. Response options were coded as a continuous variable; response items “I am not comfortable answering this question” and “I don’t know” were coded as a non-response. The first three income categories (less has $10,000; $10,000–$19,999 and $20,000–$29,999) were combined and the midpoint of $20,000 was used due to low numbers in these categories.

### Statistical methods

The statistical analysis was performed with SAS (University Edition, Version Studio, SAS Institute, Cary, NC, USA). Linear regression was used to explore the association between HEI-2015 scores and both avoidable and unavoidable daily per capita food waste (total and for each food category). Household income was included in all models. A *p*-value less than 0.05 was used to establish statistical significance.

## Results

### Sample characteristics

**Fig. 2:**
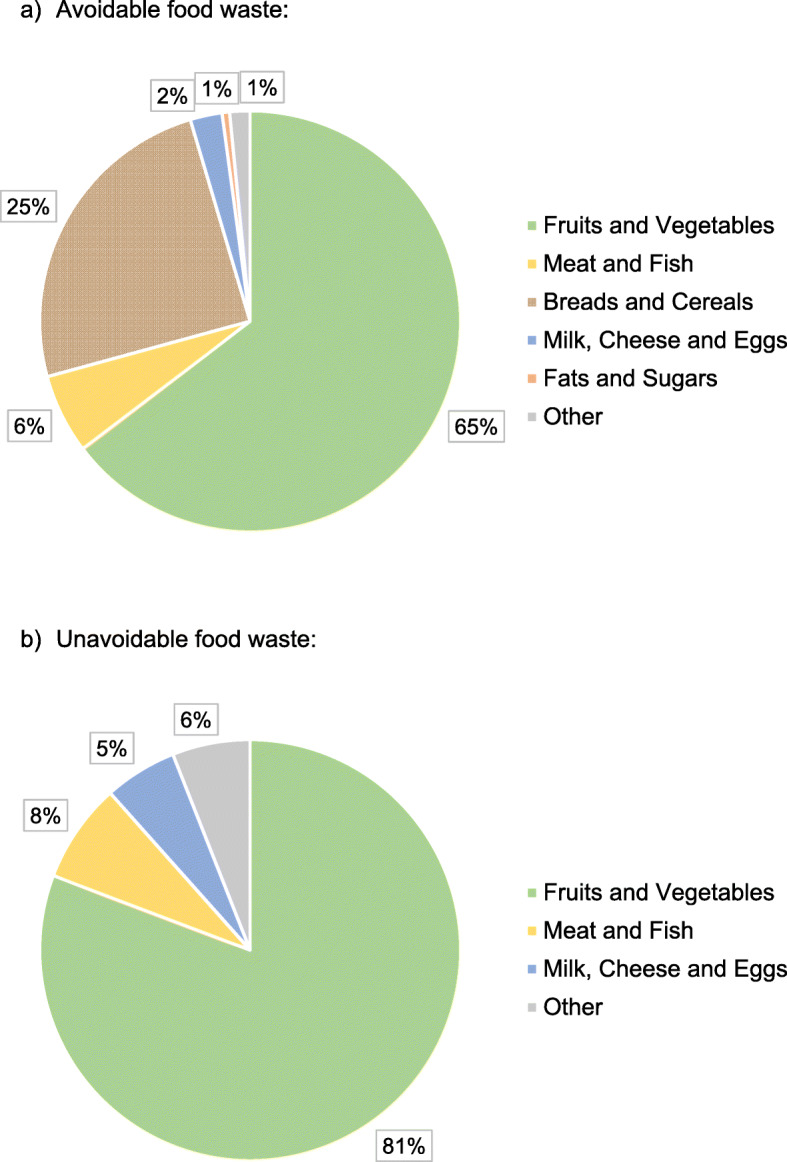
Percent daily average per capita food waste

Nearly half of the families had a household income of over $100,000 per year and 60% of participating families reported having at least 4 members in their household (Table 1). Approximately 80% of the participating parents identified as Caucasian and 85% of participating parents were mothers. Parent diet quality scores ranged from 37 to 92; 81% of parents had diet scores in the “Needs Improvement (51-80)” category and 12% had diets scores in the “Poor (≤50) category. Child diet quality scores ranged from 32 to 91; 82% of children had diet scores in the “Needs Improvement” category and 5% had diet scores in the “Poor” category. Parent and child HEI scores were significantly correlated (r = 0.61; *P <* 0.0001). Figure 2 details the distribution of food waste across participating households. On average, households produced approximately 107 g of avoidable food waste per person daily. Fruits and vegetables were the highest contributor to avoidable food waste: 65% of avoidable food waste came from fruits and vegetables. The mean unavoidable food waste per person daily was approximately 52 g.

**Table 1:** Characteristics among parents (*n* = 85) and children (*n* = 85) in the Family Food Skills Study

Variable	*n* (%)
Household Income ^a^	
*<$40,000*	9 (11%)
*$40,000-$59,999*	11 (14%)
*$60,000-$79,999*	7 (9%)
*$80,000-$99,999*	14 (18%)
*$100,000-$149,9999*	28 (35%)
*>$150,000*	10 (13%)
Parent Ethnicity	
*Caucasian*	70 (82%)
*Other*	15 (18%)
Parent Gender	
*Female*	72 (85%)
*Male*	13 (15%)
Total Family Members	
*Two*	4 (5%)
*Three*	14 (16%)
*Four*	51 (60%)
*Five*	11 (13%)
*Six+*	5 (6%)
Parent Diet Quality (HEI-2015)	
*Good Diet (>80)*	6 (7%)
*Needs Improvement (51-80)*	69 (81%)
*Poor Diet (≤50)*	11 (12%)
Child Diet Quality (HEI-2015)	
*Good Diet (>80)*	11 (13%)
*Needs Improvement (51-80)*	70 (82%)
*Poor Diet (≤50)*	4 (5%)

^a^Six parents did not disclose their household income

### Parent diet quality and daily per capita food waste

Parent HEI-2015 scores were not significantly associated with total avoidable and unavoidable daily per capita food waste (Tables 2 and 3). In analyses exploring associations between diet quality and each of the food categories, parent diet quality was positively associated with daily per capita for both avoidable (Unstandardized β = 1.05; 95%CI: 0.11, 1.99; *P* = 0.03) and unavoidable (Unstandardized β = 0.60; 95%CI: 0.03, 1.17; *P* = 0.04) fruit and vegetable waste. No significant associations were found between parent diet quality and the other food waste categories of avoidable or unavoidable waste.

**Table 2:** Association between diet quality scores and daily per capita avoidable food waste among parent participants. *n* = 79 ^b^

	Unadjusted β (95% CI)	Adjusted β (95% CI) ^a, c^
*Total Avoidable Waste*	0.81 (− 0.38, 1.99)	0.85 (− 0.43, 2.13)
*Fruits and Vegetables*	**1.06 (0.20, 1.91)**	**1.05 (0.11, 1.99)**
*Milk, Cheese and Eggs*	−0.05 (− 0.14, 0.05)	−0.009 (− 0.07, 0.05)
*Meat and Fish*	− 0.08 (− 0.24, 0.08)	−0.10 (− 0.28, 0.07)
*Breads and Cereals*	− 0.06 (− 0.47, 0.34)	−0.03 (− 0.44, 0.40)
*Fats and Sugars*	− 0.02 (− 0.05, 0.01)	− 0.01 (− 0.05, 0.02)

^a^ Model was adjusted for household income

^b^ Bold estimates are statistically significant at *p* < 0.05

^c^ β estimates are unstandardized

**Table 3:** Association between diet quality scores and daily per capita unavoidable food waste among parent participants. *n* = 79 ^b^

	Unadjusted β (95% CI)	Adjusted β (95% CI) ^a, c^
*Total Unavoidable Waste*	0.53 (− 0.02, 1.08)	0.52 (− 0.08, 1.12)
*Fruits and Vegetables*	**0.64 (0.12, 1.16)**	**0.60 (0.03, 1.17)**
*Milk, Cheese and Eggs*	0.006 (− 0.04, 0.05)	0.03 (− 0.009, 0.08)
*Meat and Fish*	−0.02 (− 0.10, 0.06)	−0.01 (− 0.10, 0.08)

^a^ Model was adjusted for household income

^b^ Bold estimates are statistically significant at *p* < 0.05

^c^ β estimates are unstandardized

### Child diet quality and daily per capita food waste

No significant associations were found between child HEI-2015 scores and total avoidable and unavoidable daily per capita food waste or between child HEI-2015 scores and any of the food waste categories, after adjusting for household income (Tables 4 and 5). In the unadjusted model, a significant positive association was observed in child diet quality and avoidable daily per capita fruit and vegetables waste.

**Table 4:** Association between diet quality scores and daily per capita avoidable food waste among child participants. *n* = 79 ^b^

	Unadjusted β (95% CI)	Adjusted β (95% CI) ^a, c^
*Total Avoidable Waste*	0.97 (− 0.27, 2.21)	0.84 (− 0.44, 2.12)
*Fruits and Vegetables*	**1.01 (0.11, 1.92)**	0.93 (− 0.007, 1.87)
*Milk, Cheese and Eggs*	0.01 (−0.09, 0.12)	0.02 (−0.04, 0.09)
*Meat and Fish*	0.03 (−0.13, 0.20)	0.03 (− 0.15, 0.21)
*Breads and Cereals*	−0.08 (− 0.51, 0.34)	−0.13 (− 0.55, 0.29)
*Fats and Sugars*	− 0.004 (− 0.04, 0.03)	−0.004 (− 0.04, 0.03)

^a^ Model was adjusted for household income

^b^ Bold estimates are statistically significant at *p* < 0.05

^c^ β estimates are unstandardized

**Table 5:** Association between diet quality scores and daily per capita unavoidable food waste among child participants. *n* = 79 ^b^

	Unadjusted β (95% CI)	Adjusted β (95% CI) ^a, c^
*Total Unavoidable Waste*	0.43 (− 0.15, 1.02)	0.37 (− 0.23, 0.97)
*Fruits and Vegetables*	0.49 (− 0.06, 1.05)	0.44 (− 0.14, 1.01)
*Milk, Cheese and Eggs*	0.02 (−0.03, 0.06)	0.02 (− 0.02, 0.07)
*Meat and Fish*	−0.006 (− 0.09, 0.08)	−0.01 (− 0.10, 0.08

^a^ Model was adjusted for household income

^b^ Bold estimates are statistically significant at *p* < 0.05

^c^ β estimates are unstandardized

## Discussion

This study explored the association between diet quality (HEI-2015) and daily per capita avoidable and unavoidable food waste among a sample of Canadian families. Although parent diet quality was not significantly associated with total per capita food waste, parent diet quality was positively associated with both daily per capita avoidable and unavoidable fruit and vegetable waste after adjusting for household income. Similarly, a significant positive association was observed in child diet quality avoidable daily per capita fruit and vegetables waste in the unadjusted models. However, after adjusting for household income no significant associations were found. Although estimates of association between children’s diet quality and food waste were similar to the estimates of association among parents, the confidence intervals were wider. Use of parent proxy reports of children’s diets likely resulted in greater error in the measurement of children’s diet, which could have led to greater variability in the children’s results.

This is the first study to explore the association between diet quality and food waste using waste audits, which provide an accurate and detailed assessment of food waste. Our approach used a thorough sorting method to measure the different types of food being discarded, as well as whether or not these food items could have been avoided. The previous study by Conrad and colleagues [3], which found that higher quality diets were associated with greater total food waste among American adults, used estimates for food waste from US government datasets. Use of these estimates may lead to an overestimation of daily per capita food waste and could explain the discrepancy between our study findings and those by Conrad and colleagues [3]. Our study found that the mean daily per capita food waste was approximately 159 g, whereas Conrad et al. [3] reported 422 g of daily per capita food waste. In our sample, 65% of avoidable food waste came from fruits and vegetables, whereas 39% of total food waste were from fruits and vegetables and mixed fruit and vegetable dishes in the study by Conrad and colleagues [3]. There are also dietary differences between Canadian and American populations which may contribute to these differences in food waste [15, 16]. Our study used 3-day food records to assess diet quality whereas Conrad et al. [3] used a single 24-h recall. Since dietary habits may differ based on the day of the week, the 3-day food record can help account for day-to-day variability.

Similar to our study, the Conrad et al. [3] findings also showed that higher quality diets were associated with greater waste of fruits and vegetables. Our results suggest that more fruits and vegetables are being consumed by parents with higher diet qualities. However, these households are also wasting more edible fruits and vegetables, implying that these healthy-eating oriented households may be purchasing more produce on a regular basis in order to enable healthy diets, although they may not be eating all of the provisioned fruits and vegetables. Some of these fruits and vegetables may be being wasted due to poor storage practices. Families may also be purchasing these foods with the intention of preparing meals at home, but their busy schedules may result in meals being purchased outside of the home, i.e., from takeout or fast food restaurants. On the other hand, parents who have lower HEI-2015 scores, might be more reliant on such convenience foods, thus resulting in less food waste being generated. Considering most Canadians eat out or purchase takeout food on a regular basis [17], it is an important habit to consider in relation to household food waste. Another possible interpretation is that substantial amounts of the edible portions of fruits and vegetables are being discarded through the food preparation process in households where a parent has higher HEI-2015 scores (again, perhaps because these households are working to provide and prepare more fruits and vegetables than households with lower diet quality). These results highlight the importance of differentiating between avoidable and unavoidable waste in household waste studies, especially with respect to fruits and vegetables, and the need for future research to identify the specific mechanisms leading to household food waste within families.

In addition to the economic and environmental impacts of food waste, waste of fruits and vegetables is also associated with the wasting of key nutrients of which many Canadians have inadequate intakes [18]. A recent analysis published by our team found that waste of avoidable fruits and vegetables was associated with substantial loss of fibre, calcium, magnesium and vitamins A and C [4]. This suggests that many families in our sample have access to fruits and vegetables but are not consuming them. In some households (and particularly among lower HEI-2015 respondents), any wastage of avoidable fruits and vegetables could contribute to inadequate intake of key nutrients. Identifying strategies to support families to efficiently prepare and consume fruits and vegetables they have purchased may help reduce levels of food waste and improve diet quality among Canadian families [19, 20].

Although our study had a number of strengths, this study also had limitations that should be considered when interpreting the results. First, our analysis included a relatively small sample of 85 families within Guelph-Wellington area of Ontario. However, given the detailed waste audits conducted, a larger sample would not have been feasible with the resources available. Second, while the waste audits were able to identify and quantify avoidable food waste among our sample, food items that are commonly discarded in the sink (e.g. fluid dairy) could not be accounted for. Third, participating households were aware of the waste audit portion of our study, which may have resulted in participants changing their waste behavior. Our study attempted to reduce this potential source of bias by collecting all three waste streams over a three to four-week period. The total waste and proportion of waste per food category in this study are similar to waste estimates found using blinded audits among households in the same region [21], suggesting that this risk of bias may be low. Fourth, the majority of our families identified as Caucasian, parents were mostly (85%) mothers and had an annual household income of over $100,000, which limits the generalizability of our results. Additional research with a diverse sample of families is needed.

While our results suggest that diet quality was not associated with total food waste, understanding predictors of food waste and identifying strategies to reduce food waste remain important. In our sample, an average of 1.91 kg of avoidable fruits and vegetables were discarded weekly and accounted for roughly 65% of avoidable household food waste. Considering that food wasted in this category was considered mostly avoidable and that diet quality scores for most parents and children fell in the “Needs Improvement (51-80)” category, future studies should test strategies designed to improve diet quality while simultaneously decreasing food waste, particularly fruits and vegetables.

## Conclusion

This is the first study to explore the association between diet quality and food waste using detailed waste audits. Our results suggest that there is no significant association between diet quality and daily per capita food waste. However, parent diet quality was positively associated with both avoidable and unavoidable fruit and vegetable waste, after adjusting for household income. Future research should explore this association among larger and more socio-economically and racially diverse samples. The relatively low quality of diets and the high level of food waste among families in our sample suggest that future research should also explore effective strategies towards improving diet quality while simultaneously reducing food waste, especially in fruits and vegetables.

## Data Availability

The datasets for this manuscript are not publicly available because of restrictions. Related to the Research Ethics Board requirements.

## Abbreviations

β: Beta
CI: Confidence interval
HEI: Healthy Eating Index
FFSS: Family Food Skills Study
*n*: Number of participants
RD: Registered Dietitian
SAS: Statistical Analysis Software
SD: Standard deviation
USDA: United States Department of Agriculture
WRAP: Waste and Resources Action Programme

## References

[1] Gooch M, Felfel A, Marenick N. Food waste in Canada: opportunities to increase the competitiveness of Canada’s agri-food sector, while simultaneously improving the environment. 2010. Available at: https://vcm-international.com/wp-content/uploads/2013/04/Food-Waste-in-Canada-112410.pdf Accessed 22 Jan 2020.

[2] Gooch M, Bucknell D, LaPlain D, Dent B, Whitehead P, Felfel A, et al. The avoidable crisis of food waste: technical report. 2019. Available at: https://secondharvest.ca/wp-content/uploads/2019/01/Avoidable-Crisis-of-Food-Waste-Technical-Report-January-17-2019.pdf Accessed 22 Jan 2020.

[3] Conrad Z, Niles MT, Neher DA, Roy ED, Tichenor NE, Jahns L (2018). Relationship between food waste, diet quality and environmental sustainability. PLoS One.

[4] von Massow M, Parizeau K, Gallant M, Wickson M, Haines J, Ma DWL, Wallace A, Carroll N, Duncan AM (2019). Valuing the multiple impacts of household food waste. Front Nutr.

[5] Bellemare MF, Çakir M, Peterson HH, Novak L, Rudi J (2017). On the measurement of food waste. Am J Agric Econ.

[6] van der Werf P, Seabrook JA, Gilliland JA. The quantity of food waste in the garbage stream of southern Ontario, Canada households. PLoS ONE. 2018;13(6). 10.1371/journal.pone.0198470 PMC5999097 29897964

[7] Secondi L, Principato L, Laureti T (2015). Household food waste behaviour in EU-27 countries: a multilevel analysis. Food Policy.

[8] Parizeau K, von Massow M, Martin R (2015). Household-level dynamics of food waste production and related beliefs, attitudes, and behaviours in Guelph. Ontario Waste Manag.

[9] Krebs-Smith SM, Pannucci TE, Subar AF, Kirkpatrick SI, Lerman JL, Tooze JA, Wilson MM, Reedy J (2018). Update of the healthy eating index: HEI-2015. J Acad Nutr Diet.

[10] Jessri M, Ng AP, L'Abbé MR (2017). Adapting the healthy eating index 2010 for the Canadian population: evidence from the Canadian National Nutrition Survey. Nutrients..

[11] Household food waste collections guide. Available at: http://www.wrap.org.uk/content/household-food-waste-collections-guide. Accessed 18 Jan 2020.

[12] Quested T, Parry A, Easteal S, Swannell R (2011). Food and drink waste from households in the UK. Nutr Bull.

[13] Setti M, Falasconi L, Segrè A, Cusano I, Vittuari M (2016). Italian consumers’ income and food waste behavior. Br Food J.

[14] French SA, Tangney CC, Crane MM, Wang Y, Appelhans BM (2019). Nutrition quality of food purchases varies by household income: the SHoPPER study. BMC Public Health.

[15] Colapinto CK, Graham J, St-Pierre S (2018). Trends and correlates of frequency of fruit and vegetable consumption, 2007 to 2014. Health Rep.

[16] Lee-Kwan SH, Moore LV, Blanck HM, Harris DM, Galuska D (2017). Disparities in state-specific adult fruit and vegetable consumption - United States, 2015. Morb Mortal Wkly Rep.

[17] Statistics Canada: Eating out – How often and why? Available at: https://www150.statcan.gc.ca/n1/pub/11-627-m/11-627-m2019003-eng.pdf. .

[18] Garriguet D (2009). Diet quality in Canada. Health Rep.

[19] Parfitt J, Barthel M, Macnaughton S (2010). Food waste within food supply chains: quantification and potential for change to 2050. Philos Trans R Soc B.

[20] Larson N, Perry C, Story M, Neumark-Sztainer D (2006). Food preparation by young adults is associated with better diet quality. J Am Diet Assoc.

[21] von Massow M, Parizeau K. Household food waste audit data in Guelph area. Unpublished raw data.

